# Association of Monoamine Oxidase A (MAOA) Gene uVNTR and rs6323 Polymorphisms with Attention Deficit and Hyperactivity Disorder in Korean Children

**DOI:** 10.3390/medicina54030032

**Published:** 2018-05-18

**Authors:** In Wook Hwang, Myung Ho Lim, Ho Jang Kwon, Han Jun Jin

**Affiliations:** 1 Environmental Health Center, Dankook Medical Hospital, Cheonan, 31116, Korea; hg1427@gmail.com (I.W.H.); paperose@dankook.ac.kr (M.H.L.); hojangkwon@gmail.com (H.J.K.); 2 Department of Biological Sciences, College of Natural Science, Dankook University, Cheonan, 31116, Korea; 3 Department of Psychology, College of Public Welfare, Dankook University, Cheonan, 31116, Korea; 4 Department of Preventive Medicine, College of Medicine, Dankook University, Cheonan, 31116, Korea

**Keywords:** ADHD, *MAOA*, BASC-2, polymorphism, Korean children

## Abstract

*Objective*: Attention deficit hyperactivity disorder (ADHD) is a common neurodevelopmental disorder. The genetic cause of ADHD is still unclear, but the dopaminergic, serotonergic, and noradrenergic pathways have shown a strong association. In particular, monoamine oxidase A (MAOA) plays an important role in the catabolism of these neurotransmitters, suggesting that the *MAOA* gene is associated with ADHD. Therefore, we evaluated the relationship between the *MAOA* gene polymorphisms (uVNTR and rs6323) and ADHD. *Materials and methods*: We collected a total of 472 Korean children (150 ADHD cases and 322 controls) using the Korean version of the Dupaul Attention Deficit Hyperactivity Disorder Rating Scales (K-ARS). Genotyping was performed by PCR and PCR-RFLP. The Behavior Assessment System for Children Second Edition (BASC-2) was used to evaluate the problem behaviors within ADHD children. *Results*: We observed significant associations between the rs6323 and ADHD in girls (*p* < 0.05) and the TT genotype was observed as a protective factor against ADHD in the recessive model (OR 0.31, 95% CI 0.100–0.950, *p* = 0.022). The 3.5R-G haplotype showed a significant association in ADHD boys (*p* = 0.043). The analysis of subtype also revealed that the 4.5R allele of uVNTR was a risk factor for the development of ADHD in the combined symptom among girls (OR 1.87, 95% CI 1.014–3.453, *p* = 0.031). In the BASC-2 analysis, the *MAOA* uVNTR polymorphism was associated with activities of daily living in ADHD boys (*p* = 0.017). *Conclusion*: These results suggest the importance of the *MAOA* gene polymorphisms in the development of ADHD in Korean children. A larger sample set and functional studies are required to further elucidate of our findings.

## 1. Introduction

Attention Deficit Hyperactivity Disorder (ADHD) is one of the most common neurodevelopmental disorders with a prevalence of 8.5% among the Korean children [1]. It is characterized by clinical symptoms of inattentive or hyperactive and impulsive behavior [2]. ADHD is a heterogeneous disorder that is possibly affected by a variety of genetic and environmental factors [3]. As with other causes, the genetic etiology of ADHD is still unclear, and many studies have reported that the genes related to the dopaminergic, serotonergic, and noradrenergic pathways are significantly associated with ADHD [4,5,6,7]. Among these genes, the *DRD4* and *DAT1* gene VNTR polymorphisms of the dopaminergic pathway, *HTR1B* gene polymorphisms (rs6296 and rs6298) of the serotonergic pathway, and *NET* gene polymorphisms (rs2242446, rs28386840, and rs5569) of the noradrenergic pathway have shown a consistent association with ADHD in the various populations [8,9,10].

The monoamine oxidase A (*MAOA*) gene is located on the X chromosome (Xp11.23) [11], and plays an important role in the catabolism of monoamine neurotransmitters including dopamine, serotonin, and norepinephrine [12]. Thus, the *MAOA* gene has been noted in a variety of neurodevelopmental disorders and behavioral studies in both animals and human subjects [13]. Kim et al. (2014) analyzed the association between *MAOA* gene polymorphisms (rs6323, rs1137070, and rs3027407) and patients with schizophrenia [14]. The study found that three SNPs were associated with common clinical symptoms in male schizophrenia patients. Lung et al. (2011) showed that a functional polymorphism in the *MAOA* gene was significantly associated with major depressive disorder (MDD) [15]. Reif et al. (2012) collected a total of 1306 panic disorder patients from clinical samples and controls from community samples for meta-analysis and found a significant association between the *MAOA* gene uVNTR and panic disorder [16]. The *MAOA* gene knockout mice showed elevated levels of dopamine, serotonin, and norepinephrine, which resulted in increased aggressive behavior [17].

In general, the relationship between the *MAOA* gene and behavior phenotypes is known to be regulated by the environmental factors including stress, alcohol dependence, physical activity, and food deprivation [18,19,20]. Kinnally et al. (2009) reported that adult women who were exposed to family stressors had a lower activity of *MAOA*, and showed higher impulsivity/aggression behaviors [21]. However, Keller et al. (2014) mentioned that environmental variables (e.g., childhood maltreatment) were modulated by a specific genetic polymorphism [22]. Likewise, the majority of phenotypes are polygenic [23], which means that studies on various polymorphisms are needed to evaluate the cause of the phenotypes such as behaviors.

Many studies have reported that the upstream variable number tandem repeats (uVNTR) in the promoter and the rs6323 of *MAOA* gene are associated with ADHD [11,24]. Recently, Karmakar et al. (2017) found that the *MAOA* gene rs6323 polymorphism was associated with behavioral problems in ADHD males in the Indian population [25]. These polymorphisms (uVNTR and rs6323) are known to be functional and affect the activities of *MAOA* enzymes [26,27]. Previously, Das et al. (2006) found that the frequency of the 3.5 repeat allele was significantly higher in ADHD cases than in the control group in the Indian population [28]. Xu et al. (2007) reported the genetic association between the *MAOA* gene rs6323 G allele and ADHD in the Taiwanese population [24]. However, these findings were inconsistent in the different populations [13,24,29,30]. Lawson et al. (2003) reported no association between *MAOA* gene polymorphisms (rs6323 and uVNTR) and ADHD in a UK population [30]. Manor et al. (2002) showed that the 4.5 repeat allele was associated with ADHD in the Israeli population [31]. Such discrepancies in the findings in the different populations need investigations in an independent population to determine the role of the *MAOA* gene polymorphisms on ADHD.

Therefore, we hypothesized that the *MAOA* gene polymorphisms (uVNTR and rs6323) will contribute to the development of ADHD and behavior phenotypes in Korean children. To assess this hypothesis, we collected a total of 472 samples (150 ADHD children, 322 controls), and used the BASC-2 behavior scales.

## 2. Materials and Methods

### 2.1. Subject

We analyzed a total of 472 samples from the Children’s Health and Environmental Research (CHEER) cohort study and a previous study described in [32]. Of these samples, 120 ADHD children, 322 control individuals were a subset of the CHEER cohort study and 30 ADHD children were a subset of the samples analyzed in Kwon et al. (2014). The CHEER study was carried out on elementary school children from 10 cities in Korea from 2005 to 2010 biennially. An interview was randomly performed on children using the Korean version of the Dupaul Attention Deficit Hyperactivity Disorder Rating Scales (K-ARS) [33] including 18 items based on the DSM-IV diagnostic criteria for ADHD. In this study, the K-ARS score for the ADHD samples were 19 or higher, and the score for the control group was less than 19. Of these 18 items, questions 1–9 were for inattention and questions and 10–18 were for hyperactive/impulsive. According to these criteria, we classified subtype of ADHD cases as follows; combined (ADHD/C, n = 78), predominantly inattentive ADHD/I, (n = 32) and predominantly hyperactive/impulsive (ADHD/HI, n = 10) [34]. Informed consent was obtained from all the participants of this study. The sex and age for the ADHD children and the control group were matched to avoid possible biases of the study. A clinical evaluation and the DSM-IV diagnosis [2] were performed by a child psychiatrist. In the ADHD children, 97 (64.7%) were boys, and 53 (35.3%) were girls, and the mean age was 8.05 ± 1.04. The control group consist of 191 boys (59.3%) and 131 girls (40.7%), and the mean age was 8.22 ± 1.48. The study protocol was approved by the Ethics Committee of Dankook University Hospital.

### 2.2. Assessment and Behavior Rating Scales

The Behavior Assessment System for Children Second Edition (BASC-2) is a standardized multi-dimensional rating system that is used in schools to evaluate of skills, adaptive behaviors, and problem behaviors by teacher, parents and/or the children themselves [35]. In this study, we used only the Parent Rating Scale (PRS). This was standardized for the three age ranges including preschoolers (ages 2–5 years), children (6–11 ages), and adolescents (12–21 years). Among these, only the child (PRS-C: 160 items) form was used. These items were classified in nine clinical scales (i.e., Aggression, Anxiety, Attention Problems, Atypicality, Conduct Problems, Depression, Hyperactivity, Somatization, and Withdrawal) and five adaptive scales (i.e., Activities of Daily Living, Adaptability, Functional Communication, Leadership, and Social Skills). Each item was rated on a four-point scale depending on frequency of occurrence (never = 0, sometimes = 1, often = 2, and almost always = 3) and the item raw scores were summed.

### 2.3. DNA Extraction and Genotyping

DNA was extracted from leukocytes using the G-DEX^TM^ llb Genomic DNA Extraction kit (Intron Biotechnology, Seongnam, Korea) or the GeneAll Exgene Clinic SV mini kit (GeneALL, Seoul, Korea). uVNTR and rs6323 were genotyped using PCR and PCR-RFLP, respectively. The primer set for the determination of the uVNTR was described by Sabol et al. (1998) [27] and the rs6323 primer set was designed using the Primer3Plus web-based tool [36]. Genomic DNA was amplified using the new designed primers as follow: forward, 5′-ACAGCCTGACCGTGGAGAAG-3′ and reverse 5′-GAACGGACGCTCCATTCGGA-3′ for uVNTR; forward, 5′-TAATTAATGCGATCCCTCCG-3′ and reverse 5′-TGAGGAAATTGACAGACCAAGA-3′ for rs6323. Each PCR reaction was performed in a total volume of 20 µL containing 10 ng of genomic DNA, 10 pM each primer, 0.2 mM dNTPs, 2.0 mM MgCl_2_, 10 × PCR buffer and 1.0 U NV DNA polymerase (NAVI BioTech, Cheonan, Korea). The PCR amplification was conducted C1000 Touch thermal cycler (Bio Rad, Hercules, USA) under the following conditions: 95 °C for 5 min, followed by 35 cycles of 94 °C for 1 min, 60 °C for 1 min, and 72 °C for 1 min, and then a final extension at 72 °C for 10 min. The PCR products of uVNTR were present for 2.5R (294 bp), 3.5R (324 bp), 4.5R (354 bp), and 5.5R (384 bp). The amplification product of rs6323 was digested with 1.0 U *Fnu4H* I restriction enzyme (New England Biolabs, Massachusetts, USA) for 8 h at 37 °C and electrophoresed in 3% agarose gel (Lonza, Morristown, NJ, USA). The polymorphic *Fnu4H* I site was detected by restriction fragment length polymorphism that produced fragments of 141, 84, 72 bp (G allele) or 156, 141 bp (T allele).

### 2.4. Data Analyses

Independent (unpaired) samples *t*-test was performed to compare the variables (i.e., age, BASC-2 scores) using the SPSS 21 Statistics software (IBM Korea, Seoul, Korea). The test of cross tabulation analyses, and odds ratio (OR) with 95% confidence intervals (CI) were calculated in a 2 × 2 table using the genotype and allele frequencies of the cases and controls in web-based statistical tools (SISA, http://www.quantitativeskills.com/sisa/ and SNPstats, http://bioinfo.iconcologia.net/snpstats). Haplotypes and linkage disequilibrium (LD) were estimated using the SNPstats web-based tool (SNPstats, https://www.snpstats.net/snpstats/). The Bonferroni correction was applied to adjust the α-level according to the number of markers (<0.05/2 = 0.025). It has been reported that approximately 210 subjects are needed to obtain a 95% sample power in a case-control study of ADHD in the Korean population [32]. In this study, we expected that there was sufficient power to evaluate the effects between the *MAOA* gene polymorphisms and ADHD.

## 3. Results

We analyzed a total of 472 Korean children. No statistical differences were observed in the value of age (F = 46.61, *p* = 0.130) and gender (F = 1.23, *p* = 0.270) between the ADHD children and the controls (Table 1). Genotype distributions of two polymorphisms in ADHD children and the control group were in agreement with the Hardy-Weinberg equilibrium (HWE) except for rs6323 in ADHD girls (*p* = 0.023) (Table 2) and ADHD-C girls (*p* = 0.033) (Table 3). Since males are hemizygous for the X chromosome, the test of Hardy-Weinberg equilibrium was not applicable in boys.

We found significant associations in the genetic models of *MAOA* gene rs6323 polymorphism between the ADHD girls and control girls (*p* < 0.05) (Table 2). Especially, the TT genotype was observed as a protective factor for ADHD in the recessive model (OR 0.31, 95% CI 0.100–0.950, *p* = 0.022). In contrast, no significant differences were observed between the *MAOA* uVNTR and incidence of ADHD (*p* > 0.05). In the subtype analysis, the G/T genotype of the rs6323 polymorphism was revealed a significant association with the development of ADHD combined symptom among girls in the over-dominant model (OR 3.01, 95% CI 1.223–7.426, *p* = 0.013) (Table 3). The 4.5R allele of uVNTR was a risk factor for ADHD combined symptom in girls (OR 1.87, 95% CI 1.014–3.453, *p* = 0.031). In addition, this trend was also observed in the analysis of genotype and dominant model (*p* < 0.05) (Table 3). These two polymorphisms (uVNTR and rs6323) were in a high LD (Supplementary Table S1); the 3.5R-G haplotype appeared to have a higher frequency in ADHD boys than the control boys (*p* = 0.043) (Table 4). Correlation analysis was performed to evaluate the relationship between BASC-2 PRS scales and *MAOA* gene polymorphisms in ADHD children. In this analysis, the *MAOA* uVNTR was significantly associated with the activities of daily living of Adaptive Scale in ADHD boys (*p* = 0.017) (Table 5). The rs6323 polymorphism was not associated with the BASC-2 PRS scales (data not shown).

## 4. Discussion

We evaluated the role of *MAOA* gene polymorphisms (uVNTR and rs6323) in the development of ADHD and investigated the effects of ADHD symptoms.

Here, we observed slight deviations from the Hardy-Weinberg equilibrium in rs6323 of ADHD girls and ADHD-C girls (*p* < 0.05). It is known that a deviation from the Hardy-Weinberg equilibrium in the case group presents a genetic association with disease [37]. Thus, it implies the significant role of the rs6323 polymorphism on the occurrence of ADHD in Korean girls. We observed significant differences in the genetic models of *MAOA* gene rs6323 polymorphism between ADHD girls and control girls (*p* < 0.05) (Table 2). The frequency of the rs6323 TT genotype was higher in the control girls than in the ADHD girls (ADHD girls: 7.5% and control girls: 20.6%) and the frequencies of the rs6323 genotype and allele in the control girls were consistent with the previous study [14]. The rs6323 was located in the exon 8 of *MAOA* gene, which makes an amino acid change from valine to phenylalanine [38]. It has been reported that the rs6323 gene polymorphism was associated with high (G allele) and low (T allele) *MAOA* enzyme activity [26]. Xu et al. (2007) found that the rs6323 G allele frequency was higher in ADHD children when compared to the controls [24]. Domschke et al. (2005) also observed a significantly increased transmission of rs6323 G allele in ADHD cases [39]. In particular, the study reported that the increased MAOA enzyme activity led to dopamine deficiency and ADHD. These findings are consistent with the general hypothesis that the imbalance of dopamine levels contribute to the development of ADHD. Therefore, our result implies that the rs6323 TT genotype would be a protective factor against ADHD in Korean girls.

For the linkage disequilibrium (LD) analysis, the standardized estimate of LD was calculated as D’ and the corresponding *p*-value [40]. We confirmed that the uVNTR and rs6323 were in LD status in our samples (Supplementary Table S1). Thus, we performed a haplotype analysis based on the LD status of the two polymorphisms. The 3.5R-G haplotype frequency was significantly higher in ADHD boys than in the control boys (*p* = 0.043) (Table 4). It has been reported that the 30bp uVNTR located 1.2kb upstream of the *MAOA* coding sequence regulates the transcriptional activity of the *MAOA* gene [27]. Previously, the short allele (3R) of uVNTR showed low enzyme activity and was associated with ADHD [41,42]. Xu et al. (2007) reported the significant association between the 3R-G haplotype and ADHD in the Taiwanese population [24]. According to Das et al. (2006), the 3.5 and 4.5 repeats of *MAOA* uVNTR corresponded to the three and four repeats, respectively [28]. Thus, our results support the previous results where the 3.5R (or 3R) of uVNTR and G allele of rs6323 were significantly associated with ADHD.

We performed the subtype (combined, inattentive, and hyperactive/impulsive) analysis of ADHD. Inattentive (I) and Hyperactive-Impulsive (HI) subtypes were not analyzed due to the small sample size (Table 3). In the combined subtype (C), we observed genetic associations between the *MAOA* gene polymorphisms (uVNTR and rs6323) and ADHD girls (*p* < 0.05). Previously, Brookes et al. (2006) reported the association between SNPs of the *MAOA* gene and ADHD-combined subtype [43]. The 4.5R of uVNTR is more frequent in ADHD-C girls. This finding was inconsistent with a previous report where the 3.5R of uVNTR was significantly associated with ADHD. Some genetic association studies of ADHD have suggested that the ADHD subtypes were influenced by distinct genetic factors from ADHD [44,45]. This means that additional analysis is required with more samples to validate the effect of 4.5R of uVNTR in ADHD-C girls.

Merrell (2008) suggested the BASC-2 scales as one of the best tools for diagnosing behavioral disorder [46]. Among these BASC-2 scales, the activities of daily living scale of Adaptive Scale were provided to diagnose the adaptive behavior deficits and cognitive functioning in children and adolescents [35]. It is also known that this behavioral measure is related to acting in a safe manner, performing simple daily tasks, creating ideas, and social sufficiency [47,48]. Recently, we reported the genetic correlation of BASC-2 in ADHD boys [49]. In this study, the activities of daily living of Adaptive Scale in ADHD boys was significantly associated with *MAOA* uVNTR (*p* < 0.05). Many studies have reported that the *MAOA* uVNTR polymorphism was related to various behaviors such as aggression, impulsivity and antisocial behaviors in males [42,50,51]. In particular, the 3R (short allele) of *MAOA* uVNTR was a risk factor for antisocial behavior [52]. These findings were consistent with our result. However, the genetic correlation between *MAOA* uVNTR and BASC-2 scales is still unclear. Therefore, additional functional studies can help to find the underlying evidence for our results.

There were some important limitations to this study. First, some significant results did not survive after the Bonferroni correction. However, several studies have reported that with the strict statistical threshold of the Bonferroni method, it would be possible to miss significant effects [53]. Second, the sample size was relatively small. Thus, we calculated the statistical power of our samples for this study. We obtained a sample power of more than 90%. Suresh and Chandrashekara et al. (2012) reported that a sufficient sample power for clinical research was approximately 80% [54]. Third, we did not consider sample characteristics such as socioeconomic level and parental marital status. In general, these sociodemographic characteristics are known to be important in the development of ADHD [1]. Therefore, additional studies will need to consider these variables for acquiring more exact results. Finally, the 150 ADHD children were recruited through different origin (120 cases from the CHEER cohort, and another 30 from a previous study). However, since they were collected by the same child psychiatrist with the same diagnostic criteria (DSM-IV), we believe that the sampling bias would not be a main issue in our study.

In contrast to the limitations, our study also has certain advantages. First, our subjects were matched for sex and age to adjust the effects of these variables. Second, this study used population-based samples. The total subjects were selected by a questionnaire survey from the whole population within the main 10 cities in Korea through the CHEER cohort study and sampling were completely random. Thus, it can be said that the samples can represent the Korean population. Third, all the sample individuals were chosen using the clinical evaluation and DSM-IV diagnosis by child psychiatrists. It strictly distinguished the ADHD children and control groups. Therefore, our ADHD children samples can be defined as “pure ADHD” without comorbidity.

## 5. Conclusions

In summary, *MAOA* gene polymorphisms were shown to be associated with the development of ADHD and behavioral traits. In particular, we observed that the rs6323 TT genotype was a protective factor against ADHD in Korean children. The *MAOA* uVNTR polymorphism was associated with the activities of daily living on BASC-2 behavior scales. Therefore, our results imply that the *MAOA* gene polymorphisms may provide a significant effect on the occurrence of ADHD. However, since ADHD is influenced by various variables, further studies are needed on larger sample sizes that consider these variables in order to clarify our findings.

## Figures and Tables

**Table 1:** Descriptive data about sample (control and ADHD) characteristics.

Characteristics		ADHD (n = 150) ^a^	Control (n = 322)	F or χ^2^	*p*-value
Age ^b^		8.05 ± 1.04	8.22 ± 1.48	46.61	0.13 ^b^
Gender	Boys	97 (64.7%)	191 (59.3%)	1.23	0.27 ^c^
	Girls	53 (35.3%)	131 (40.7%)		
Nationality		Republic of Korea	Republic of Korea		

^a^ CHEER study 120 ADHD samples + 30 ADHD samples analyzed in Kwon et al. (2014); ^b^ These data represent mean ± SD, by Independent *t*-test; ^c^ The Chi-square *p*-value.

**Table 2:** Comparison of allele and genotype frequencies of two *MAOA* gene polymorphisms between ADHD children and controls.

	Allele/Genotype	ADHD-Boys	Control-Boys	*p*-Value ^a^	OR (95% CI)
Rs6323	G	62 (63.9%)	102 (53.4%)	0.088	0.64 (0.391–1.069)
	T	35 (36.1%)	89 (46.6%)		
uVNTR	3.5R	64 (66.0%)	109 (58.0%)	0.190	0.71 (0.427–1.185)
	4.5R	33 (34.0%)	79 (42.0%)		
	Allele/genotype	ADHD-girls	Control-girls	*p*-value ^a^	OR (95% CI)
Rs6323	G	65 (61.3%)	152 (58.0%)	0.559	0.87 (0.549–1.393)
	T	41 (38.7%)	110 (42.0%)		
	G/G	16 (30.2%)	48 (36.7%)	-	Reference
	G/T	33 (62.3%)	56 (42.7%)	0.114	1.77 (0.869–3.598)
	T/T	4 (7.5%)	27 (20.6%)	0.175	0.44 (0.135–1.465)
	*p*-value ^b^	0.023 *	0.161		
Dominant	G/G	16 (30.2%)	48 (36.6%)	0.400	0.75 (0.380–1.480)
	G/T+T/T	37 (69.8%)	83 (63.4%)		
Recessive	G/G+G/T	49 (92.5%)	104 (79.4%)	0.022 *	0.31 (0.100–0.950)
	T/T	4 (7.5%)	27 (20.6%)		
Over-dominant	G/G+T/T	20 (37.7%)	75 (57.2%)	0.016 *	2.21 (1.150–4.250)
	G/T	33 (62.3%)	56 (42.8%)		
uVNTR	3.5R	62 (60.8%)	150 (59.5%)	0.826	1.05 (0.658–1.687)
	4.5R	40 (39.2%)	102 (40.5%)		
	3.5R/3.5R	17 (33.3%)	48 (38.1%)	-	Reference
	3.5R/4.5R	28 (54.9%)	54 (42.9%)	0.296	0.68 (0.330–1.400)
	4.5R/4.5R	6 (11.8%)	24 (19.0%)	0.515	1.42 (0.490–4.060)
	*p*-value ^b^	0.279	0.214		
Dominant	3.5R/3.5R	17 (33.3%)	48 (38.1%)	0.550	0.81 (0.410–1.610)
	3.5R/4.5R+4.5R/4.5R	34 (66.7%)	78 (61.9%)		
Recessive	3.5R/3.5R+3.5R/4.5R	45 (88.2%)	102 (81.5%)	0.230	1.76 (0.680–4.610)
	4.5R/4.5R	6 (11.8%)	24 (19.1%)		
Over-dominant	3.5R/3.5R+4.5R/4.5R	23 (45.1%)	72 (57.1%)	0.150	0.62 (0.320–1.190)
	3.5R/4.5R	28 (54.9%)	54 (42.9%)		

* *p* < 0.05; ^a^ The Chi-square *p*-value; ^b^ Hardy-Weinberg equilibrium *p*-value. The samples who have 2.5 or 5.5 repeats of uVNTR were excluded because of the small sample size.

**Table 3:** Comparison of allele and genotype frequencies of two *MAOA* gene polymorphisms in the ADHD combined subtype and controls.

	Allele/Genotype	ADHD-C ^a^ Boys (n = 52)	Control Boys (n = 191)	*p*-Value ^b^	OR (95% CI)
Rs6323	G	34 (65.4%)	102 (53.4%)	0.123	0.61 (0.321–1.149)
	T	18 (34.6%)	89 (46.6%)		
uVNTR	3.5R	34 (65.4%)	109 (58.0%)	0.335	0.73 (0.385–1.386)
	4.5R	18 (34.6%)	79 (42.0%)		
	Allele/genotype	ADHD-C ^a^ girls (n = 26)	Control girls (n = 131)	*p*-value ^b^	OR (95% CI)
Rs6323	G	30 (57.7%)	152 (58.0%)	1.000	1.01 (0.555–1.851)
	T	22 (42.3%)	110 (42.0%)		
	G/G	6 (23.1%)	48 (36.7%)	-	Reference
	G/T	18 (69.2%)	56 (42.7%)	0.058	2.57 (0.945–6.998)
	T/T	2 (7.7%)	27 (20.6%)	0.535	0.59 (0.112–3.143)
	*p*-value ^b^	0.033 *	0.161		
Dominant	G/G	6 (23.1%)	48 (36.6%)	0.183	0.52 (0.195–1.381)
	G/T+T/T	20 (76.9%)	83 (63.4%)		
Recessive	G/G+G/T	24 (92.3%)	104 (79.4%)	0.121	0.32 (0.071–1.443)
	T/T	2 (7.7%)	27 (20.6%)		
Over-dominant	G/G+T/T	8 (31.8%)	75 (57.2%)	0.013 *	3.01 (1.223–7.426)
	G/T	18 (69.2%)	56 (42.8%)		
uVNTR	3.5R	22 (44.0%)	150 (59.5%)	0.031 *	1.87 (1.014–3.453)
	4.5R	28 (56.0%)	102 (40.5%)		
	3.5R/3.5R	3 (12.0%)	48 (38.1%)	-	Reference
	3.5R/4.5R	16 (64.0%)	54 (42.9%)	0.009 *	4.74 (1.301–17.273)
	4.5R/4.5R	6 (24.0%)	24 (19.0%)	0.050	4.00 (0.920–17.397)
	*p*-value ^b^	0.135	0.214		
Dominant	3.5R/3.5R	3 (12.0%)	48 (38.1%)	0.011 *	4.51 (1.282–15.889)
	3.5R/4.5R+4.5R/4.5R	22 (88.0%)	78 (61.9%)		
Recessive	3.5R/3.5R+3.5R/4.5R	19 (76.0%)	102 (81.5%)	0.571	1.34 (0.484–3.721)
	4.5R/4.5R	6 (24.0%)	24 (19.1%)		
Over-dominant	3.5R/3.5R+4.5R/4.5R	9 (36.0%)	72 (57.1%)	0.053	2.37 (0.974–5.770)
	3.5R/4.5R	16 (64.0%)	54 (42.9%)		

Inattentive (I) and Hyperactive-Impulsive (HI) subtypes were not analyzed due to the small sample size. * *p* < 0.05. ^a^ Combined subtype. ^b^ Uncorrected chi-square or Fisher’s test, as appropriate. ^c^ Hardy-Weinberg equilibrium *p*-value. The samples who have 2.5 or 5.5 repeats of uVNTR were excluded because of the small sample size.

**Table 4:** Comparison of *MAOA* gene polymorphisms (rs6323 and uVNTR) haplotype frequencies between ADHD children and controls.

Gender	Haplotype	Overall	Case	Control	*p*-Value
Boys	3.5-G	0.530	0.608	0.489	0.043 *
	3.5-T	0.354	0.309	0.378	0.246
	4.5-G	0.077	0.052	0.090	0.258
	4.5-T	0.039	0.031	0.043	0.528
Girls	3.5-G	0.536	0.531	0.537	0.900
	3.5-T	0.063	0.077	0.058	0.377
	4.5-G	0.060	0.087	0.050	0.468
	4.5-T	0.341	0.305	0.355	0.189

* *p* < 0.05.

**Table 5:** Association between the *MAOA* uVNTR and BASC-2 score of ADHD boys.

BASC-2 ^a^	Category	*MAOA* uVNTR		*p*-Value ^b^
Clinical Scale		3.5R (n = 56)	4.5R (n = 22)	
	Hyperactivity	7.25 ± 3.38	7.55 ± 3.64	0.734
	Aggression	5.11 ± 3.32	5.14 ± 3.50	0.973
	Anxiety	9.23 ± 4.33	10.05 ± 6.47	0.591
	Depression	5.39 ± 4.04	6.23 ± 3.01	0.384
	Somatization	3.35 ± 3.12	3.32 ± 3.21	0.973
	Atypicality	4.20 ± 3.87	4.36 ± 2.87	0.858
	Conduct Problems	4.13 ± 2.89	5.00 ± 2.90	0.232
	Attention Problems	8.27 ± 3.32	9.45 ± 3.17	0.154
	Withdrawal	7.29 ± 3.85	7.05 ± 3.80	0.804
Adaptive Scale				
	Adaptability	13.68 ± 4.44	12.09 ± 3.32	0.134
	Social Skills	10.88 ± 3.71	10.18 ± 4.26	0.479
	Leadership	10.39 ± 4.00	8.77 ± 4.87	0.135
	Activities of Daily Living	13.18 ± 3.36	11.14 ± 3.24	0.017 *
	Functional Communication	23.40 ± 6.04	22.05 ± 5.44	0.364

^a^ Behavior Assessment System for Children Second Edition (Reynolds and Kamphaus, 2004). ^b^ These data represent mean ± SD, by Independent *t*-test. * *p* < 0.05.

## Supplementary Materials

The following are available online at http://www.mdpi.com/1648-9144/54/3/32/s1.

## References

[1] Kim M.J., Park I., Lim M.H., Paik K.C., Cho S., Kwon H.J., Lee S.G., Yoo S.J., Ha M. (2017). Prevalence of Attention-Deficit/Hyperactivity Disorder and its Comorbidity among Korean Children in a Community Population. J. Korean Med. Sci..

[2] American Psychiatric Association Committee on Nomenclature and Statistics (1994). Diagnostic and Statistical Manual of Mental Disorders (DSM-IV).

[3] Tarver J., Daley D., Sayal K. (2014). Attention-deficit hyperactivity disorder (ADHD): An updated review of the essential facts. Child Care Health.

[4] Faraone S.V. (2004). Genetics of adult attention-deficit/hyperactivity disorder. Psychiatr. Clin. North.

[5] Franke B., Neale B.M., Faraone S.V. (2009). Genome-wide association studies in ADHD. Hum. Genet..

[6] Schachar R. (2014). Genetics of Attention Deficit Hyperactivity Disorder (ADHD): Recent Updates and Future Prospects. Curr. Dev. Disord. Rep..

[7] Akutagava-Martins G.C., Rohde L.A., Hutz M.H. (2016). Genetics of attention-deficit/hyperactivity disorder: An update. Expert Rev. Neurother..

[8] Kirley A., Lowe N., Hawi Z., Mullins C., Daly G., Waldman I., McCarron M., O’Donnell D., Fitzgerald M., Gill M. (2003). Association of the 480 bp DAT1 allele with methylphenidate response in a sample of Irish children with ADHD. Am. J. Med. Genet. B Neuropsychiatr. Genet..

[9] Ickowicz A., Feng Y., Wigg K., Quist J., Pathare T., Roberts W., Malone M., Schachar R., Tannock R., Kennedy J.L. (2007). The serotonin receptor HTR1B: Gene polymorphisms in attention deficit hyperactivity disorder. Am. J. Med. Genet. B Neuropsychiatr. Genet..

[10] Oh S.Y., Kim Y.K. (2016). Association of norepinephrine transporter gene polymorphisms in attention-deficit/hyperactivity disorder in Korean population. Prog. Neuropsychopharmacol. Biol. Psychiatry.

[11] Liu L., Guan L.L., Chen Y., Ji N., Li H.M., Li Z.H., Qian Q.J., Yang L., Glatt S.J., Faraone S.V. (2011). Association analyses of MAOA in Chinese Han subjects with attention-deficit/hyperactivity disorder: Family-based association test, case-control study, and quantitative traits of impulsivity. Am. J. Med. Genet. B Neuropsychiatr. Genet..

[12] Qiu H.T., Meng H.Q., Song C., Xiu M.H., Zhu F.Y., Wu G.Y., Kosten T.A., Kosten T.R., Zhang X.Y. (2009). Association between monoamine oxidase (MAO)-A gene variants and schizophrenia in a Chinese population. Brain Res..

[13] Qian Q.J., Liu J., Wang Y.F., Yang L., Guan L.L., Faraone S.V. (2009). Attention Deficit Hyperactivity Disorder comorbid oppositional defiant disorder and its predominately inattentive type: Evidence for an association with COMT but not MAOA in a Chinese sample. Behav. Brain Funct..

[14] Kim S.K., Park H.J., Seok H., Jeon H.S., Chung J.H., Kang W.S., Kim J.W., Im Yu G., Shin D.H. (2014). Association study between monoamine oxidase A (MAOA) gene polymorphisms and schizophrenia: Lack of association with schizophrenia and possible association with affective disturbances of schizophrenia. Mol. Biol. Rep..

[15] Lung F.W., Tzeng D.S., Huang M.F., Lee M.B. (2011). Association of the MAOA promoter uVNTR polymorphism with suicide attempts in patients with major depressive disorder. BMC Med. Genet..

[16] Reif A., Weber H., Domschke K., Klauke B., Baumann C., Jacob C.P., Ströhle A., Gerlach A.L., Alpers G.W., Pauli P. (2012). Meta-analysis argues for a female-specific role of MAOA-uVNTR in panic disorder in four European populations. Am. J. Med. Genet. B Neuropsychiatr. Genet..

[17] Chen K., Holschneider D.P., Wu W., Rebrin I., Shih J.C. (2004). A spontaneous point mutation produces monoamine oxidase A/B knock-out mice with greatly elevated monoamines and anxiety-like behavior. J. Biol. Chem..

[18] Lavigne J.V., Herzing L.B., Cook E.H., Lebailly S.A., Gouze K.R., Hopkins J., Bryant F.B. (2013). Gene × environment effects of serotonin transporter, dopamine receptor D4, and monoamine oxidase A genes with contextual and parenting risk factors on symptoms of oppositional defiant disorder, anxiety, and depression in a community sample of 4-year-old children. Dev. Psychopathol..

[19] Voltas N., Aparicio E., Arija V., Canals J. (2015). Association study of monoamine oxidase-A gene promoter polymorphism (MAOA-uVNTR) with self-reported anxiety and other psychopathological symptoms in a community sample of early adolescents. J. Anxiety Disord..

[20] Naoi M., Riederer P., Maruyama W. (2016). Modulation of monoamine oxidase (MAO) expression in neuropsychiatric disorders: Genetic and environmental factors involved in type A MAO expression. J. Neural Transm..

[21] Kinnally E.L., Huang Y.Y., Haverly R., Burke A.K., Galfalvy H., Brent D.P., Oquendo M.A., Mann J.J. (2009). Parental care moderates the influence of MAOA-uVNTR genotype and childhood stressors on trait impulsivity and aggression in adult women. Psychiatr. Genet..

[22] Keller M.C. (2014). Gene × environment interaction studies have not properly controlled for potential confounders: The problem and the (simple) solution. Biol. Psychiatry.

[23] Fowler T., Langley K., Rice F., van den Bree M.B., Ross K., Wilkinson L.S., Owen M.J., O’donovan M.C., Thapar A. (2009). Psychopathy trait scores in adolescents with childhood ADHD: The contribution of genotypes affecting MAOA, 5HTT and COMT activity. Psychiatr. Genet..

[24] Xu X., Brookes K., Chen C.K., Huang Y.S., Wu Y.Y., Asherson P. (2007). Association study between the monoamine oxidase A gene and attention deficit hyperactivity disorder in Taiwanese samples. BMC Psychiatry.

[25] Karmakar A., Goswami R., Saha T., Maitra S., Roychowdhury A., Panda C.K., Sinha S., Ray A., Mohanakumar K.P., Rajamma U. (2017). Pilot study indicate role of preferentially transmitted monoamine oxidase gene variants in behavioral problems of male ADHD probands. BMC Med. Genet..

[26] Hotamisligil G.S., Breakefield X.O. (1991). Human monoamine oxidase A gene determines levels of enzyme activity. Am. J. Hum. Genet..

[27] Sabol S.Z., Hu S., Hamer D. (1998). A functional polymorphism in the monoamine oxidase A gene promoter. Hum. Genet..

[28] Das M., Das Bhowmik A., Sinha S., Chattopadhyay A., Chaudhuri K., Singh M., Mukhopadhyay K. (2006). MAOA promoter polymorphism and attention deficit hyperactivity disorder (ADHD) in Indian children. Am. J. Med. Genet. B Neuropsychiatr. Genet..

[29] Hawi Z., Matthews N., Barry E., Kirley A., Wagner J., Wallace R.H., Heussler H.S., Vance A., Gill M., Bellgrove M.A. (2013). A high density linkage disequilibrium mapping in 14 noradrenergic genes: Evidence of association between SLC6A2, ADRA1B and ADHD. Psychopharmacology.

[30] Lawson D.C., Turic D., Langley K., Pay H.M., Govan C.F., Norton N., Hamshere M.L., Owen M.J., O’Donovan M.C., Thapar A. (2003). Association analysis of monoamine oxidase A and attention deficit hyperactivity disorder. Am. J. Med. Genet. B Neuropsychiatr. Genet..

[31] Manor I., Tyano S., Mel E., Eisenberg J., Bachner-Melman R., Kotler M., Ebstein R.P. (2002). Family-based and association studies of monoamine oxidase A and attention deficit hyperactivity disorder (ADHD): Preferential transmission of the long promoter-region repeat and its association with impaired performance on a continuous performance test (TOVA). Mol. Psychiatry.

[32] Kwon H.J., Jin H.J., Lim M.H. (2014). Association between monoamine oxidase gene polymorphisms and attention deficit hyperactivity disorder in Korean children. Genet. Test. Mol. Biomark..

[33] Kim Y.S., So Y.K., Noh J.S., Ko S.G., Koh Y.J. (2002). The reliability and validity of Korean parent and teacher ADHD Rating Scale. J. Korean Neuropsychiatr. Assoc..

[34] DuPaul G.J., Anastopoulos A.D., McGoey K.E., Power T.J., Reid R., Ikeda M.J. (1997). Teacher ratings of attention deficit hyperactivity disorder symptoms: Factor structure and normative data. Psychol. Assess..

[35] Reynolds C.R., Kamphaus R.W. (2004). Behavior Assessment for Children, (BASC-2).

[36] Untergasser A., Nijveen H., Rao X., Bisseling T., Geurts R., Leunissen J.A. (2007). Primer3Plus, an enhanced web interface to Primer3. Nucleic Acids Res..

[37] Ogus A.C., Yoldas B., Ozdemir T., Uguz A., Olcen S., Keser I., Coskun M., Cilli A., Yegin O. (2004). The Arg753GLn polymorphism of the human toll-like receptor 2 gene in tuberculosis disease. Eur. Respir. J..

[38] Liu Z., Huang L., Luo X.J., Wu L., Li M. (2016). MAOA Variants and Genetic Susceptibility to Major Psychiatric Disorders. Mol. Neurobiol..

[39] Domschke K., Sheehan K., Lowe N., Kirley A., Mullins C., O’Sullivan R., Freitag C., Becker T., Conroy J., Fitzgerald M. (2005). Association analysis of the monoamine oxidase A and B genes with attention deficit hyperactivity disorder (ADHD) in an Irish sample: Preferential transmission of the MAO-A 941G allele to affected children. Am. J. Med. Genet. B Neuropsychiatr. Genet..

[40] Zill P., Büttner A., Eisenmenger W., Möller H.J., Bondy B., Ackenheil M. (2004). Single nucleotide polymorphism and haplotype analysis of a novel tryptophan hydroxylase isoform (TPH2) gene in suicide victims. Biol. Psychiatry.

[41] Deckert J., Catalano M., Syagailo Y.V., Bosi M., Okladnova O., Di Bella D., Nöthen M.M., Maffei P., Franke P., Fritze J. (1999). Excess of high activity monoamine oxidase A gene promoter alleles in female patients with panic disorder. Hum. Mol. Genet..

[42] Manuck S.B., Flory J.D., Ferrell R.E., Mann J.J., Muldoon M.F.A. (2000). regulatory polymorphism of the monoamine oxidase-A gene may be associated with variability in aggression, impulsivity, and central nervous system serotonergic responsivity. Psychiatry Res..

[43] Brookes K., Xu X., Chen W., Zhou K., Neale B., Lowe N., Aneey R., Franke B., Gill M., Ebstein R. (2006). The analysis of 51 genes in DSM-IV combined type attention deficit hyperactivity disorder: Association signals in DRD4, DAT1 and 16 other genes. Mol. Psychiatry..

[44] Guan L., Wang B., Chen Y., Yang L., Li J., Qian Q., Wang Z., Faraone S.V., Wang Y. (2009). A high-density single-nucleotide polymorphism screen of 23 candidate genes in attention deficit hyperactivity disorder: Suggesting multiple susceptibility genes among Chinese Han population. Mol. Psychiatry.

[45] Shang C.Y., Gau S.S., Liu C.M., Hwu H.G. (2011). Association between the dopamine transporter gene and the inattentive subtype of attention deficit hyperactivity disorder in Taiwan. Prog. Neuropsychopharmacol. Biol. Psychiatry.

[46] Merrell K.W. (2008). Behavioral, Social, and Emotional Assessment of Children and Adolescents.

[47] Kamphaus R.W., Reynold C.R., Kamphaus R.W. (2003). Assessment of adaptive behavior. Handbook of Psychological and Educational Assessment of Children.

[48] Harrison J.R., Vannest K.J., Reynolds C.R. (2011). Behaviors that discriminate ADHD in children and adolescents, primary symptoms, symptoms of comorbid conditions, or indicators of functional impairment?. J. Atten. Disord..

[49] Hwang I.W., Hong J.H., Kwon B.N., Kim H.J., Lee N.R., Lim M.H., Kwon H.J., Jin H.J. (2017). Association of mitochondrial DNA 10398 A/G polymorphism with attention deficit and hyperactivity disorder in Korean children. Gene.

[50] Sjöberg R.L., Nilsson K.W., Wargelius H.L., Leppert J., Lindström L., Oreland L. (2007). Adolescent girls and criminal activity: Role of MAOA-LPR genotype and psychosocial factors. Am. J. Med. Genet. B Neuropsychiatr. Genet..

[51] Buckholtz J.W., Meyer-Lindenberg A. (2008). MAOA and the neurogenetic architecture of human aggression. Trends Neurosci..

[52] Ficks C.A., Waldman I.D. (2014). Candidate genes for aggression and antisocial behavior: A meta-analysis of association studies of the 5HTTLPR and MAOA-uVNTR. Behav. Genet..

[53] Zhao Y., He A., Zhu F., Ding M., Hao J., Fan Q., Li P., Liu L., Du Y., Liang X. (2018). Integrating genome-wide association study and expression quantitative trait locus study identifies multiple genes and gene sets associated with schizophrenia. Prog. Neuropsychopharmacol. Biol. Psychiatry.

[54] Suresh K., Chandrashekara S. (2012). Sample size estimation and power analysis for clinical research studies. J. Hum. Reprod. Sci..

