# Supplementary material for: Association of Monoamine Oxidase A (MAOA) Gene uVNTR and rs6323 Polymorphisms with Attention Deficit and Hyperactivity Disorder in Korean Children
Source: Medicina (Kaunas). 2018 May 18;54(3):32. doi: 10.3390/medicina54030032 (PMC6122096; doi:10.3390/medicina54030032)
Supplement: Supplementary file 1 [file medicina-54-00032-s001.pdf]

Supplementary Table S1. Pairwise Linkage Disequilibrium (LD) Matrices in the ADHD children and controls.

Group (Boys)	VNTR	G941T	Frequency	D	D´
Case					
	3.5	G	0.608	0.187	0.858
	3.5	T	0.309		
	4.5	G	0.052		
	4.5	T	0.031		
Control					
	3.5	G	0.489	0.181	0.810
	3.5	T	0.378		
	4.5	G	0.090		
	4.5	T	0.043		

Group (Girls)	VNTR	G941T	Frequency	D	D´
Case					
	3.5	G	0.531	0.155	0.668
	3.5	T	0.077		
	4.5	G	0.087		
	4.5	T	0.305		
Control					
	3.5	G	0.537	0.188	0.789
	3.5	T	0.058		
	4.5	G	0.050		
	4.5	T	0.355		
